# Surgical Management of Septic Arthritis of the Wrist: An Analysis of Short- and Long-Term Functional and Clinical Outcomes

**DOI:** 10.3390/life15030372

**Published:** 2025-02-26

**Authors:** Yonca Steubing, Felix Reinkemeier, Flemming Puscz, Sonja Verena Schmidt, Jannik Hinzmann, Marcus Lehnhardt, Mehran Dadras

**Affiliations:** 1 Department of Plastic Surgery, Burn and Sarcoma Center, Hand Surgery, BG University Hospital Bergmannsheil Bochum, Ruhr-University Bochum, 44789 Bochum, Germany; 2 Department of Plastic, Reconstructive and Aesthetic Surgery, Agaplesion Diakonieklinikum Hamburg, 20259 Hamburg, Germany

**Keywords:** septic arthritis, wrist, arthrotomy, arthroscopy, hand surgery

## Abstract

Septic arthritis of the wrist is a rare but severe condition requiring urgent diagnosis and treatment to prevent joint destruction and functional impairment. The objective of this study was to investigate prognostic parameters and the long-term functional outcomes. This retrospective and prospective cohort study included 44 patients treated for septic arthritis of the wrist between 2008 and 2024. All patients underwent surgical wrist arthrotomy due to concomitant soft tissue involvement, with a median follow-up of 29 months. Clinical outcomes were assessed through total active range of motion (TROM), grip strength, and patient-reported hand function using the DASH questionnaire. Data analysis examined correlations between comorbidities, surgical interventions and long-term outcomes. *Staphylococcus aureus* was identified in 61.4% of cases, thus being the most common pathogen. Type 2 diabetes mellitus was identified as a risk factor for requiring a higher number of surgeries to control the infection. Patients requiring more invasive procedures showed worse long-term outcomes, with lower grip strength, a limited active range of motion (TROM) and higher DASH scores. Mortality was associated with higher inflammatory markers and older age at the onset of disease. A total of 36.4% of patients were unable to return to work after treatment, while those who successfully returned to work experienced a median recovery period of seven months. The timely initiation of appropriate surgical therapy is essential in the treatment of septic arthritis of the wrist in order to reduce joint damage and associated loss of hand function, particularly in patients with comorbidities such as diabetes mellitus. Studies should focus on comparing different treatment options and developing more targeted rehabilitation strategies to improve functional outcomes.

## 1. Introduction

Septic arthritis of the wrist is a rare condition but represents a clinical emergency in hand surgery due to the potential for joint damage. The clinical presentation includes a painful wrist, potentially accompanied by redness, warmth, swelling and a restricted range of motion. The underlying causes may include conditions such as gout, activated osteoarthritis, crystal arthropathies, cellulitis or bacterial arthritis, with the etiology being atraumatic or trauma-associated [1]. The diagnosis is challenging, as inflammation markers and radiographs do not correlate with the severity of the infection [2,3].

In the diagnostic process, patient history must be considered, including pre-existing joint damage, infections in other joints and anatomic locations, prior trauma and, in particular, medical interventions such as intravenous lines or surgeries in the hand region. In a study assessing the prevalence of septic arthritis of the wrist in patients with atraumatic wrist pain accompanied by erythema or swelling, the condition was identified in only 5% of cases, indicating a relatively low prevalence [1]. However, the prevalence was notably higher in individuals with a history of trauma or prior interventions involving the hand or wrist region [4]. The most common pathogens are *Staphylococcus aureus* and *Streptococcus* spp. [5].

The preferred treatment for suspected septic arthritis, based on the clinical presentation and the patient’s overall health, involves collecting microbiological samples through joint aspiration or surgical arthrotomy. Following this, an empiric antibiotic therapy should be initiated [5,6]. Unlike other larger joints, the aspiration of the wrist is more challenging and may result in false-negative results, with the potential of a delay in the initiation of the appropriate therapy [7]. Delaying therapy can lead to irreversible joint damage, which is associated with long-term functional impairments [8]. The standard treatment includes arthroscopic or open drainage and lavage of the wrist as well as intravenous antibiotic therapy [6].

Despite the potential consequences, there is limited literature on management of septic arthritis of the wrist. To address this gap, a retrospective analysis of patients treated for wrist septic arthritis was conducted and combined with follow-up examinations. The aim of this study was to identify prognostic parameters and evaluate the long-term functional outcomes of this rare but impactful condition.

## 2. Patients and Methods

Patients with wrist septic arthritis who were treated between 2008 and 2024 at our institution were identified based on medical records. The inclusion criterion was a positive microbiological finding in the intraoperative biopsy, while patients under the age of 18 and those with additional infectious sources were excluded.

The retrospective analysis of patient, disease and treatment data was conducted using records from the hospital’s internal documentation system. The recruited patients were invited to a one-time follow-up appointment at our clinic after providing consent for the study. The examination methods included active range of motion of the wrists for extension/flexion, radial/ulnar deviation and pronation/supination according to the Neutral-0 Method, the measurement of grip strength using a dynamometer, and the DASH (Disabilities of the Arm, Shoulder and Hand) questionnaire. The study was conducted according to the Declaration of Helsinki, and approval by the Ethics Committee of the Medical Association of Westphalia-Lippe was obtained (approval number 2024-252-f-S).

The statistical analysis was performed using IBM SPSS (version 29.0). After testing for normality using the Shapiro–Wilk Test, group comparisons for the normally distributed variables were conducted using the independent t-test, and for non-normally distributed variables, the Kruskal–Wallis Test for independent samples was applied. Post hoc pairwise comparisons following the Kruskal–Wallis Test for >3 groups were corrected using the Bonferroni method. For the correlation analysis, Spearman’s rank correlation test (Spearman’s Rho) was used. A *p*-value of <0.05 was considered statistically significant. The values are presented as means with standard deviations for normally distributed data and as medians with interquartile ranges (IQRs) for non-normally distributed data.

## 3. Results

A total of 44 patients were included in the study, with a mean age of 65.4 years and a median follow-up of 29 months. A total of 32 patients (72.7%) were male, and 12 were female (27.2%). Of the 44 identified patients, 12 died during the follow-up period, and in 9 cases, no follow-up could be obtained. Of the 12 (22.7%) deceased patients, 6 died due to sepsis during their hospital stay, between one and fourteen days after surgery, while the remaining 6 patients died within one to three years after the operation. Four patients had known medication- or cancer-induced immunosuppression (9%), one patient had gout (2.3%), one patient had rheumatoid arthritis (2.3%), and eight patients had type 2 diabetes mellitus (18.2%). Of the septic arthritis cases, 12 (27.3%) were trauma-associated, due to cuts/stab wounds from work or gardening (9) and animal bites (3). Nine (22.5%) were iatrogenic, resulting from vascular access on the dorsum of the hand (5), postoperative complications (2) or corticosteroid injections in the hand region (2). In the remaining 23 cases (52.3%), no trauma or interventions had been reported (see Table 1).

**Figure 1:**
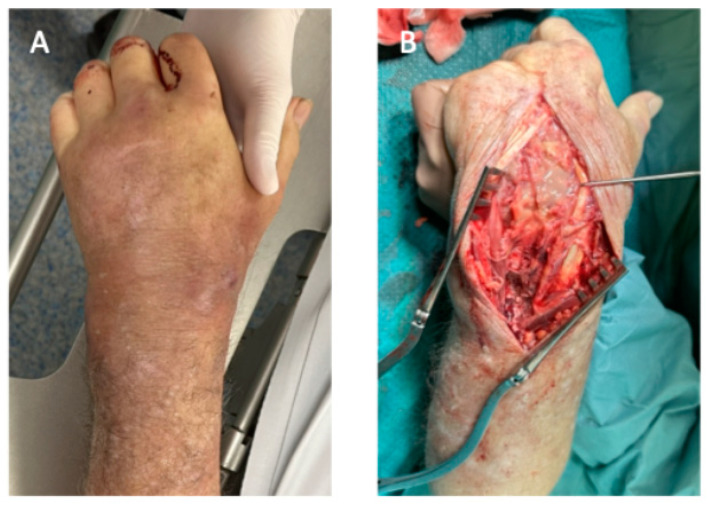
Case presentation of an 82-year-old patient with fulminant septic arthritis of the wrist. (**A**) The clinical presentation of the patient with swelling, warmth and loss of wrist function. Upon inquiry, no trauma to the hand was recalled, and the clinical examination revealed no injuries to the soft tissue envelope. (**B**) The intraoperative findings after dorsal incision and wrist arthrotomy revealed putrid secretion.

Almost all patients exhibited multiple signs of inflammation at presentation at our hospital, with 92.5% presenting pain, swelling and loss of function, 82% presenting redness, and 59% localized warmth (see Figure 1). Fever (>38 °C) was diagnosed in 31.8% of patients. The median symptom duration before admission was 4.5 days (IQR 12). Prior treatment had been administered in 36% of cases: 5% through immobilization, 20.5% with antibiotics and 10.3% via surgical intervention.

Upon admission, patients had a median leukocyte count of 11.7/nl and C-reactive protein (CRP) level of 13.9 mg/dL. A total of 57.5% had elevated leukocyte levels (>10.2/nL), and 97.5% had elevated CRP levels (>0.5 mg/dL). In joint fluid culture, *Staphylococcus aureus* was identified in 61.4% of cases (25 Methicillin-sensitive, 2 Methicillin-resistant), a mixed infection was found in 12% and, in the remaining cases, pathogens such as *Streptococcus dysgalactiae* (4.5%), *Streptococcus pyogenes* (4.5%), *Mycobacterium tuberculosis* (2.3%) and others were detected. In cases requiring the resection of bone structures, osteomyelitis was histopathologically confirmed in 23.5% of patients. In 17.5%, abnormalities such as osteolysis were detectable in the preoperative radiographs.

All patients were treated with surgical wrist arthrotomy, as all included patients had the additional involvement of the soft tissue envelope, making a purely arthroscopic approach insufficient. The required extent of surgery was stratified into three categories: 59.1% underwent wrist arthrotomy with debridement of the soft tissue envelope; 20.5% required the additional partial resection of the joint surfaces and external fixation for spontaneous partial arthrodesis or planned secondary arthrodesis; and another 15.9% underwent the resection of carpal bones with internal arthrodesis (see Table 1). On average, 3.1 surgeries were required. In purulent arthritis, a second-look operation was performed on the second postoperative day. For empiric antibiotic therapy, Ampicillin and Sulbactam 3 g were issued intravenously three times daily as the standard. Dose reduction was applied in cases of renal insufficiency. The antibiotic therapy was adjusted based on the results of the antibiogram. Septic patients underwent additional diagnostics to rule out other infection sources, including urine tests, chest radiography, transthoracic echocardiography, and dental examinations. Soft tissue reconstruction was necessary in 43.2% of patients; of these, 15.9% received a skin graft, 6.7% underwent local flap surgery and 20.5% received a free flap. The average duration of hospital stay was 20.4 days (SD 11.3).

### 3.1. Analysis of Prognostic Variables

#### 3.1.1. Age at Onset and Mortality

Deceased patients were significantly older at the time of disease onset, with a mean age of 77.2 years (SD 12.5), compared to surviving patients, who had a mean age of 64.1 years (SD 12.5) (*p* = 0.35).

#### 3.1.2. Comorbidities and Number of Required Surgeries

Among the analyzed comorbidities, only type 2 diabetes mellitus showed a significant association with the number of surgeries required to treat the infection and restore the soft tissue envelope, which was attributable to wound healing disorders. Patients with type 2 diabetes mellitus underwent a median of 3 surgeries (IQR 2), whereas non-diabetic patients required 2 surgeries (IQR 2) (*p* = 0.039).

### 3.2. Long-Term Outcomes Based on Number and Extent of Surgeries

**Figure 2:**
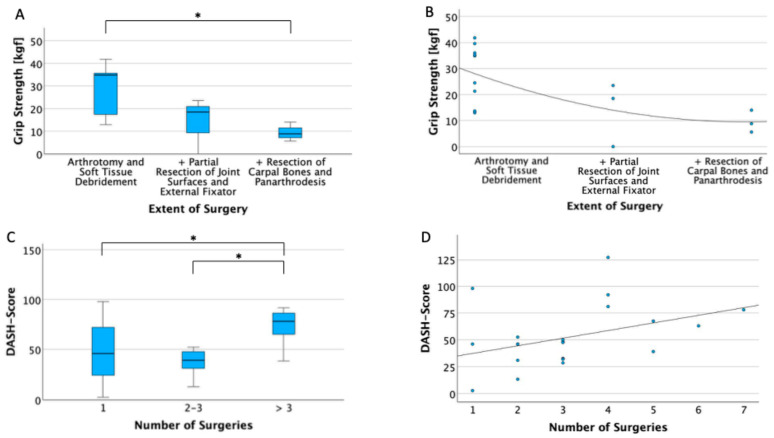
Long-term outcomes based on the number and extent of surgeries. (**A**) An illustration of grip strength (measured in kilogram-force [kgf]) on the y-axis in relation to the extent of surgical intervention on the x-axis with a box plot. The asterisks (*) indicate a significant difference between the Arthrotomy and Soft Tissue Debridement and Resection of Carpal Bones and Panarthrodesis groups (*p* = 0.034, Bonferroni-corrected). (**B**) The results of Spearman’s correlation analysis of grip strength (y-axis) and the extent of surgery (x-axis) using a scatter plot. Correlation coefficient ρ = −0.6, *p* = 0.012. (**C**) A presentation of Disabilities of the Arm, Shoulder and Hand (DASH) score results (y-axis) in relation to the number of performed surgical procedures on the x-axis using a box plot diagram. The asterisks (*) indicate a significant difference between the 1 surgery and >3 surgeries groups (*p*-value = 0.024, Bonferroni-corrected) and 2–3 surgeries and >3 surgeries groups (*p*-value = 0.028, Bonferroni-corrected). (**D**) The results of Spearman’s correlation analysis between the DASH score on the y-axis and the number of surgeries on the x-axis. The analysis shows a positive correlation between DASH score and number of surgical procedures, with a correlation coefficient of ρ = 0.64. * = *p* < 0.05.

An analysis of grip strength in the affected hand showed a statistically significant reduction in grip strength with increasing surgical invasiveness, with a *p*-value of 0.034 (Bonferroni-corrected). The grip strength of the affected side was 71.7% (SD 42.6) of the unaffected wrist. A significant negative correlation with both the extent of surgery (Spearman’s Rho ρ = −0.6, *p* = 0.012) and the number of required surgeries was observed (ρ = −0.64, *p* = 0.006). Subjective hand function, as assessed using the DASH questionnaire, revealed a significant increase in DASH scores in patients who underwent more than three surgeries (*p* = 0.024 between 1 and >3 surgeries and *p* = 0.028 between 2 and 3 surgeries and >3 surgeries, Bonferroni-corrected). Additionally, a positive correlation was found between the number of surgeries and DASH score (*p* = 0.042, ρ = 0.44) (see Figure 2).

**Figure 3:**
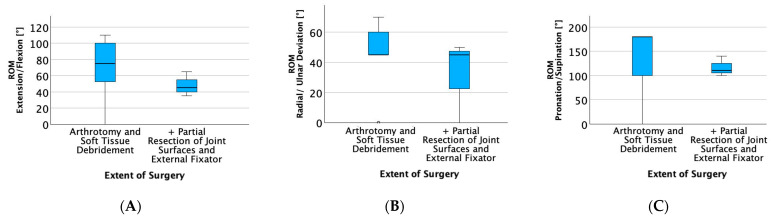
The analysis of long-term total range of motion (TROM). Boxplots illustrating the total range of motion (TROM) for (**A**) extension/flexion, (**B**) radial/ulnar deviation and (**C**) pronation/supination, each depicted on the y-axis, in degrees depending on the extent of surgeries on the x-axis. The arthrodesis group was excluded from this analysis as the surgical procedure inherently eliminates motion for extension/flexion and radial/ulnar deviation.

In the analysis of long-term active total range of motion (TROM) and pain at rest and during exertion (measured on a numerical rating scale 1–10) within the groups, the average TROM for extension/flexion (E/F) was 66.5° (SD 31.0), for radial/ulnar deviation (R/U) was 48.5° (SD 19.4) and for pronation/supination (P/S) was 143.5° (SD 62) in the group undergoing only arthrotomy and soft tissue debridement. In contrast, for patients who underwent the additional resection of joint surfaces, the average TROM for E/F was 50° (SD 21.1), for R/U was 25° (SD 35.4) and for P/S was 125° (SD 21.2) (see Figure 3). The arthrodesis group was excluded from this analysis as the surgical procedure inherently eliminates motion for extension/flexion and radial/ulnar deviation. The data demonstrate a trend towards a reduced TROM with more invasive surgical procedures, particularly after the partial resection of joint surfaces combined with external fixation. Regardless of surgical treatment, patients showed a long-term range of motion for E/F of the affected wrist of 53.3% (SD 36.7), R/U of 65.2% (SD 46.8) and P/S of 88% (SD 26.4) compared to the unaffected wrist. The results, while showing noticeable trends, did not reach statistical significance. Regarding pain, the arthrotomy group reported a mean of 0.5 (SD 1.3) at rest and 1.7 (SD 2.5) during exertion. In the group undergoing additional resection of joint surfaces, the reported pain at rest was 1.25 (SD 1.1) and during exertion, pain was 6.25 (SD 3.2). In contrast, patients who underwent wrist arthrodesis reported pain at rest as 1 (SD 1.6) and during exertion as 2 (SD 2.5).

Regarding occupational rehabilitation, 22.7% of patients were already retired prior to the condition. Of the remaining 77.3% who were still working, 40.9% returned to work, corresponding to 52.9% of those who were not retired, with a median recovery time of 7 months (IQR 4.8). Additionally, 36.4% were unable to resume work.

## 4. Discussion

Septic arthritis of the wrist is a rare condition that poses diagnostic challenges even for experienced physicians and surgeons, with significant risk for long-term functional impairment. This study offers a comprehensive perspective by analyzing both retrospective and prospective data with a median follow-up period of 29 months.

In our patient cohort, septic arthritis of the wrist was treated with the need for invasive surgery, including resection of joint surfaces or carpal bones, in 36.4% of cases, and defect coverage of the soft tissue envelope in 43.2% of cases. The lengths of stays were prolonged, with an average of 20.4 days. In the analysis, an older age of an average of 77.2 years was associated with a higher mortality risk. The presence of type 2 diabetes mellitus was found as a risk factor for the need for multiple operations to treat infections and cover defects. An analysis of the long-term outcomes revealed that greater surgical invasiveness was associated with reduced grip strength and range of motion in the affected hand, as well as higher DASH scores. Overall, only 52.9% of the patients who were still working returned to work.

The delayed initiation of therapy has been shown to have a negative impact on long-term outcomes and might increase the radicality of the required surgery [8,9]. Particularly in older patients with comorbidities such as type 2 diabetes mellitus or a history of trauma or interventions involving the hand, septic arthritis should be considered as a differential diagnosis. These findings are confirmed by a prospective study of 4907 patients conducted by Kaandorp et al., which identified independent risk factors for the development of septic arthritis, including an age of 80 years or older, diabetes mellitus, rheumatoid arthritis, recent joint interventions, skin infections and the presence of hip or knee prostheses [10].

### 4.1. Diagnostic Challenges

Laboratory tests and radiographic diagnostics demonstrate low sensitivity in the diagnosis of septic wrist arthritis [3,11]. Nevertheless, certain laboratory parameters can serve as indicators, as Roberts et al. identified elevated serum CRP levels of 10.5 mg/dL as a predictor of septic arthritis [12]. In the evaluation of diagnostic parameters in joint aspirates, studies emphasize the diagnostic value of microbiological cultures, synovial glucose levels below 28 mg/dL and lactate concentrations greater than 10 mmol/L for diagnosis [3,13,14,15]. Wrist aspiration can be difficult due to the joint’s small size and false-negative biopsies, as well as prior antibiotic treatment which may interfere with pathogen identification [1]. For this reason, antibiotics should only be administered after the collection of microbiological samples [2]; however, in cases of septic shock, antibiotics must be initiated as soon as possible, and their administration should not be delayed while awaiting surgery.

### 4.2. Treatment Strategies

As in other studies, *S. aureus* was found to be the most common pathogen in septic arthritis [1,6,16]. However, rare and atypical pathogens, including mycobacteria, pathogens associated with sexually transmitted diseases or with intravenous drug use, can also be causative [11]. When selecting antibiotic therapy, the patient’s medical history should be considered, particularly regarding the presence of atypical or resistant pathogens associated with other pre-existing conditions or prior hospitalizations [17].

In their systematic review, Mathews et al. [13] demonstrated that in four out of five studies, arthroscopic or open surgical approaches were employed. Only in a study by Goldenberg et al. from 1975 [18] were repeated needle aspirations performed, which resulted in better post-interventional functional outcomes compared to open surgery. However, the mortality rate in patients treated with needle aspirations was more than twice as high as in those who underwent surgical intervention (12% vs. 5%), and the results did not reach statistical significance. Sammer et al. reported better outcomes with fewer follow-up surgeries and shorter hospital stays for patients treated arthroscopically versus those undergoing open surgery [6]. Patients who underwent open surgical treatment required an average of three surgical interventions and had an average hospital stay of 24 days, while arthroscopically treated patients had one surgery and an average stay of 16 days [6]. In our cohort, the average number of surgeries was also three, with an average hospital stay of 20.4 days. In our hospital, all patients were surgically treated within six hours of presentation in cases of suspected septic arthritis. In all cases, an open arthrotomy was performed, as all patients presented signs of an accompanying soft tissue infection. In contrast to the cohort reported by Sammer et al. [6], whose collective predominantly included septic arthritis cases caused by hematogenous spread or cellulitis, 47.7% of our cases were post-traumatic or iatrogenic, and 10.3% had previously undergone surgical treatment at external institutions without clinical improvement. Therefore, the patient cohorts are not directly comparable. In cases of fulminant purulent joint fluid, a second-look procedure was routinely performed. Meier and Lanz [4] describe a similar approach [4]. Depending on the severity of the condition, either an arthroscopic or open surgical approach is recommended [4]. Severe infections often require partial resection of the joint capsule or ligamentous and bony structures, either immediately or during the course of treatment. In such cases, the use of an external fixator or even wrist arthrodesis may be required. In summary, the current literature suggests less invasive methods, like a combination of wrist aspiration and antibiotic therapy or arthroscopic drainage, to minimize the risk of additional long-term damage from extensive surgical interventions [6,13,19].

### 4.3. Functional Outcomes

Our analysis revealed that 36.4% of patients required partial or total joint resection due to the lysis and necrosis of the affected structures, demonstrating the severity of infection within this cohort. The combination of a severe infection and invasive surgery showed a significant decline in subjective and objective hand function. A significant negative correlation was observed between the number and extent of surgeries and grip strength, with more than three surgeries resulting in poorer subjective function measured by the DASH score, as also demonstrated in the study by Rashkoff et al. [8]. Furthermore, the need for joint surface resection was associated with reduced long-term wrist mobility and higher pain levels. These findings align with the results reported in the current literature [6,8,20] and highlight the need to carefully balance infection control with functional preservation. The results emphasize the importance of individualized rehabilitation programs to restore grip strength, range of motion and fine motor skills. Overall, there is limited literature on the long-term outcomes of septic wrist arthritis. A study of Rashkoff et al. from 1983 [8] excluded patients with osteomyelitis and postoperative septic arthritis, while the study of Hariri et al. from 2013 had a small sample size of nine patients who were only followed up 32 weeks postoperatively [20], highlighting the need of a comparative study of the short- and long-term outcomes of patients treated with either an open or arthroscopic arthrotomy.

### 4.4. Socioeconomic Impact

With 36.4% of patients unable to return to work, septic arthritis of the wrist poses substantial socioeconomic challenges. The prolonged median recovery time of seven months for those who resumed employment exhibits significant implications for both patients and healthcare systems. Notably, studies on wrist arthrodesis, another salvage procedure, report a return-to-work rate of 62.5% [21], suggesting that the acute and destructive nature of septic arthritis, combined with extensive surgical interventions, may contribute to the lower reintegration rates observed in this study. These findings emphasize the necessity of improved diagnostic protocols and early intervention strategies to reduce long-term functional impairments and enable faster reintegration into the workforce.

### 4.5. Study Limitations

The limitations of this study include the relatively small study sample, due to the rarity of wrist septic arthritis. Another potential attrition bias is the exclusion of patients who deceased or were unable to attend the clinical follow-up. These patients were mostly elderly with comorbidities, many of whom lived in nursing homes, which may have impeded their participation in the optional follow-up assessments. Additionally, the study’s inclusion criteria, which focused on cases with explicit positive microbiological findings, may have shifted the sample toward more severe presentations. The condition’s severity and the observed high mortality rate may limit the ability to generalize results to less severe cases.

## 5. Conclusions

This study underscores the complexity of diagnosing and managing septic arthritis of the wrist, as it often requires invasive surgical interventions and can result in long-term functional impairments. Older age was associated with a higher risk of mortality, and type 2 diabetes mellitus was identified as a risk factor with an increased need for additional surgeries. Our findings support the importance of early therapeutic intervention to prevent joint destruction and preserve wrist function. Extensive surgeries with the resection of joint surfaces showed a significant long-term decline in grip strength, range of motion and DASH scores. While less-invasive approaches such as arthrocentesis and arthroscopic interventions show potential for reducing surgical morbidity, the choice of therapy should be guided by the severity of the initial findings.

Future research should prioritize prospective analyses comparing the long-term outcomes of regular joint aspiration, arthroscopic procedures, and open surgical treatments. The conduct of such studies is challenging due to the rarity and heterogeneous presentation of wrist septic arthritis, but remains essential for refining clinical guidelines.

## Figures and Tables

**Table 1:** Demographic characteristics (patient, disease and treatment).

	Total Patients (*n* = 44)	Patients with Follow-Up (*n* = 23)
Age		
<60	17	11
60–80	16	8
>80	11	3
Sex		
Male	32	15
Female	12	8
Comorbidities		
Diabetes Mellitus Type 2	8	4
Immunosuppression	4	2
Rheumatoid Arthritis	1	0
Gout	1	1
Etiology		
Atraumatic	23	11
Post-traumatic	12	8
Iatrogenic	9	4
Pathogen		
Methicillin-sensitive S. aureus (MSSA)	25	13
Methicillin-resistant S. aureus (MRSA)	2	1
Other	17	9
Extent of Surgery		
Arthrotomy and Soft Tissue Debridement	26	14
and Partial Resection of Joint Surfaces and External Fixator	9	5
and Resection of Carpal Bones and Panarthrodesis	7	3
Forearm Amputation	2	1
Number of Surgeries		
1	7	5
2–3	25	12
>3	12	6
Soft Tissue Reconstruction		
Primary Closure	25	13
Skin Graft	7	5
Local Flap	3	2
Free Flap	9	3

The data are presented as absolute values.

## Data Availability

The data supporting the reported results are not publicly available due to privacy restrictions.
